# Proton Radiation Therapy for Head and Neck Cancers

**DOI:** 10.7759/cureus.70752

**Published:** 2024-10-03

**Authors:** Muhammad Sami, Muhammad Yousuf, Qasim Hashmi, Muneeb Ahmad, Mohammad Ghilman, Huzaifa Shareef

**Affiliations:** 1 Medicine, Kingston Hospital, London, GBR; 2 Haematology, Civil Hospital Karachi, Karachi, PAK; 3 Otolaryngology, Ruth K. M. Pfau, Civil Hospital Karachi, Karachi, PAK; 4 Psychiatry, Jinnah Hospital, Lahore, PAK; 5 Medicine, Dow University of Health Sciences, Civil Hospital Karachi, Karachi, PAK

**Keywords:** head and neck neoplasms, intense modulated radiation therapy (imrt), photon therapy, proton therapy, tumour cells

## Abstract

Head and neck (HnN) cancers are among the most common cancers in the world. Proton therapy (PT) is one of the latest advancements in the treatment modalities of cancers. Proton therapy is specifically used to treat HnN cancer patients due to its less toxic effects on the surrounding critical structures. Keeping in view the opportunities for further advancements, there is a lot of literature covering PT in HnN cancer patients. However, few compiled studies are not enough to compare the toxicities, overall survival (OS), local control (LC), and quality of life (QoL) of PT with that of intensity-modulated radiation therapy (IMRT). The objective of this review is to compile and summarize the literature available on the toxicities, OS, LC, and QoL in HnN cancer patients post PT. We have gathered and summarized the literature found under the keyword "proton therapy for head and neck cancers". Proton therapy is a preferable option over IMRT because it isolates tumors of the HnN, reduces exposure of healthy cells to radiation, and allows accurate tumor scanning using the pencil beam technique. In view of this article, we can say that PT is a preferable mode of radiotherapy for HnN cancer patients in view of its accuracy and lower incidents of acute and late toxicities.

## Introduction and background

Among all the malignancies, head and neck (HnN) cancers are considered the 24^th^ most common cancer in the world, as per Global Cancer Observatory (GLOBOCAN) 2022 data. It has a significantly high mortality rate, especially in developing countries [1]. Most HnN cancers arise from the mucosal epithelium in the viscera of the HnN and are collectively referred to as HnN squamous cell carcinoma (HNSCC) [2]. Of all the major culprits leading to such an outbreak of HNSCCs are tobacco and alcohol, which vary with countries and regions [2]. Apart from these, there has also been an increase in the incidence of human papillomavirus (HPV)-related oropharyngeal cancers (OPCs). Previously, HnN cancers were only presented with late-stage disease courses, but since the advent of American Joint Cancer Committee (AJCC) guidelines and patient awareness, the treatment modality has been modified [2]. 

As far as treatment options are concerned, before the advent of chemotherapy and chemo-radiation, surgery and radiation therapy were considered the primary treatment modalities for HnN cancers. The younger generation is presenting with OPCs due to the causes discussed above [3]. But in the early 1900s, surgical intervention with postoperative radiotherapy was considered, but in the late 1900s, cancer patients underwent induction chemotherapy followed by radiotherapy, then surgery [3]. 

Previously, intensity-modulated radiation therapy (IMRT) and volumetric arcs have shown promising results.  Proton therapy (PT), in contrast, is a modern form of radiotherapy with an exceptional ability to target tumor cells with maximum accuracy. Proton therapy is a preferable option over IMRT because a) it isolates tumors of the HnN, non-small cell lung cancers, and certain GI cancers where traditional photon therapy is not applicable due to overwhelming risks [1]; b) reduced exposure of healthy cells to radiation because of almost zero exit dose and accurate tumor scanning using pencil beam technique [2]. However, variations also exist in the literature; some say IMRT is better than PT, but in our observation, PT is showing better results. 

In this review, we will compare the side effects, toxicities, and limitations of IMRT as compared to proton therapy. 

## Review

Reference search 

We did not conduct a systematic review of the literature on PT in patients with HnN cancer. However, we have gathered and summarized the literature found under the keyword "proton therapy for head and neck cancers" across several renowned journals. Moreover, we also incorporated studies on reirradiation of recurrent HnN cancers and traditional photon radiotherapy in HnN cancer patients to do a comparative analysis. 

Applications, planning, delivery, and precautions of PT in HnN cancers 

Proton therapy is a safer alternative to traditional radiotherapy using photons to treat several cancers. Among the leading cases of cancer, PT is considered a preferable treatment option in HnN cancer patients due to the fact that photon therapy or IMRT cannot be used due to its toxic effects on the normal tissues of adjacent critical organs. 

Proton therapy is preferable due to its fewer effects on the surrounding healthy tissues and higher effects on the tumor. This is made possible by making doses rise sharply at the end of their penetration range and quickly fall to 0 beyond the range called Bragg Peak. Proton therapy is planned for the patient based on the tumor's location, size, grading, and tolerance. In order to get the maximum results from PT, polyenergetic beams of protons are combined to create a spread-out Bragg peak (SOBP). However, SOBP always comes with an increased risk of skin dose due to the scattering of the beam [4]. Another benefit of PT over IMRT is that PT has a near-zero exit dose, resulting in significantly reduced chances of exit point toxicity. Moreover, PT has a definite and modifiable range and penetration.  

Proton therapy can be delivered via two modes: passive scatter and intensity-modulated proton therapy (IMPT). In the former, the vertical and horizontal spread of the proton beam is regulated by adjusting the shape of its aperture. However, in IMPT, advanced magnetic pencil beam scanning allows radiologists to design a pattern of the proton beams with variable energy so as to target tumors of irregular shape and spare healthy tissues with maximum accuracy. Intensity-modulated proton therapy is the most advanced modification of PT that allows PT with minimum acute and late toxicities and increases the relative biological effectiveness (RBE) of protons [5]. 

Among the challenges faced by oncoradiologists while delivering PT effectively is delivering the Bragg peak to the desired location with maximum accuracy. Moreover, accurate delivery of PT is also affected by dental or surgical artifacts, one of the reasons radiologists prefer not to include the oral cavity in the path of the protons. Other standard operating procedures (SOPs) include minimizing the path and keeping in consideration the changes in the patient’s position, change of weight, or tumor size [6]. 

Management of different HnN cancers with PT

Cancers of Paranasal Sinuses and Nasal Cavity

Out of all HnN cancers, nasal cavity and paranasal sinus-related malignancies constitute a small fraction. Amongst all these rare malignancies, the maxillary sinus is the most commonly involved structure out of all paranasal sinuses; approximately 60%-70% of cases belong to this group [7]. Craniofacial surgery and subsequent radiotherapy remain the mainstay of treatment in most cases [8]. Even after such treatment approaches, the treatment facing inefficacy at the site of the tumor is the major concern threatening the survival of the patients [9]. However, a relationship has been made between the radiation dose and improved local control (LC), but it occurs at the cost of critical surrounding normal structures. 

The presence of optic structures in close proximity to the cancers involving paranasal sinuses and skull base; radiation therapy for these cancers results in radiation-induced retinopathy/optic neuropathy, which is frequently reported. As in research carried out at the University of Florida, the visual/ocular toxicity of radiation therapy became evident as 27% of the patients receiving radiation therapy faced unilateral blindness (radiation-induced retinopathy/optic neuropathy) and 5% suffered from bilateral blindness (optic retinalopathy) [10]. Initially, IMRT appeared to be the solution for this increased radiation-induced toxicity, but as far as LC and survival of the patients are concerned, no improvement was reported [11].

Proton therapy has shown some promising results in this regard. A study was conducted between 1991 and 2002 where 102 patients who were suffering from sinonasal cancers of different histological types and late-stage types were given PT at the Massachusetts General Hospital (MGH). The five-year LC was reported to be 86% with a mean follow-up of 6.6 years. Twenty percent of the patients received proton beam therapy (PBT) after undergoing complete resection. The median dose was set to be 71.6 Gy. For neuroendocrine, adenoid cystic, and squamous cell carcinomas, the primary relapsing pattern was distant metastasis. These consequences showed that PBT had an edge over IMRT [12]. However, when hyperfractionated proton-photon therapy was given to a group of patients at MGH, late visual and ocular toxicity was observed. Patients were followed up at a median period of 52.44 months, with only 5.6% of patients developing late-grade 3 visual/ocular toxicity [13]. No other complications were reported. After all these results, it is apparent that PBT delivers an appropriate tumoricidal dosage with the fewest complications. 

Oropharyngeal Cancer

One of the most common malignancies of the HnN includes cancers involving the oropharynx, which is the sixth most common among all malignancies [14]. As of 2008, 223,000 deaths were reported due to oral cavity and OPCs [15]. Among the etiologic factors, alcohol and tobacco are the most dominant ones. Other than these, the involvement of HPV infection has also been reported [16]. 

While treating patients suffering from OPC, radiation therapy is often preferred over surgical resection, as the latter is associated with significant functional impairment [17]. However, in 2005, Slater and colleagues conducted the first study regarding the use of proton therapy for OPCs [18]. Twenty-nine patients suffering from stage II-IV OPC were treated with combined photon-proton therapy. With 3D conformal photon being predominant (50.4 Gy), an additional (25.5 Gy) proton therapy was also given to the patients. Local control and survival without disease were 88% and 65%, respectively, in five years of follow-up. As for toxicity, it was noticed that about 16% of the patients experienced grade 3 or higher toxicity. Another study was conducted by Gun and colleagues in which it was observed that when 50 patients, the majority of whom were HPV-positive, were given PT, survival without progression was 89% in two years of follow-up [19]. None of the patients reported grade 4 or higher toxicity. However, incidences of grade 3 acute mucositis and dysphagia were reported. 

In a nutshell, it can be stated that proton beam therapy has a slight edge over IMRT, but further trials are still required to explain its role in alleviating toxicity when treating oropharyngeal cancer.

Adenoid Cystic Carcinoma (ACC)

Adenoid cystic carcinoma is a malignancy of glands that is quite rare. Several studies have suggested proton therapy with an acceptable ratio of toxicity in the cases of ACC. Adenoid cystic carcinoma of the salivary glands accounts for only 1% of the tumors of the HnN. Ammad Ud Din et al. conducted a comparative study where 23 patients suffering from ACC were given combined radiotherapy of photons and protons [20]. Varying percentages of patients had tumors involving the sinuses [21]. The extent of surgery was limited with biopsy in 48% of the patients, partial resection in 39%, whereas total resection with positive margins was done in only 13%. The median dose of proton/photon radiotherapy was 75.9 Gy, whereas the median follow-up time for surviving patients was 64 months. 

The LC rate and overall survival (OS) at five years were found to be 93% and 77%, respectively. Moreover, five years of disease-free survival and freedom from distant metastasis were found to be 56% and 62%, respectively [22]. 

One of the latest studies on treating ACC with PT by Pelak et al. involved 35 patients, out of whom 26 underwent surgery while nine were suffering from an inoperable disease [22]. The two-year LC and OS were 92.2% and 88.8%, respectively. Late and acute toxicities above grade 2 were found in 6.1% and 14.3% of the cases. Pelak et al. concluded that PBT is a useful and less toxic alternative to radiotherapy for patients with ACC. However, the patient’s age group, tumor stage, and clinical stage had a negative impact on the LC and OS ratio. The major cause of treatment failure was distant metastasis.

Nasopharyngeal Cancer (NPC)

Nasopharyngeal cancer is a common malignancy of HnN. Unlike others, its occurrence is influenced by geography. The carcinoma has its origin in the epithelium lining the nasopharynx. The WHO has categorized it into three types on a histopathological basis (a), non-keratinizing (b), and undifferentiated carcinoma (c) [23]. Nasopharyngeal cancer has a diverse etiology, as a complex mechanism leads to its clinical presentation, including Epstein-Barr virus (EBV) infection, genetic susceptibility, and environmental factors as the major culprits.  

In 2007, a study was published by Kam et al. that revealed that when IMRT was compared with 2D-radiation therapy as a treatment option for patients with uncomplicated NPC, at one year, decreased incidence of severe xerostomia rated by physicians was reported after IMRT [24]. In the late 90s, a combination of proton-photon radiation was given to patients suffering from advanced NPC at the MGH [25]. The median dose was 73.6 Gy. As far as recurrence is concerned, only one of them faced local recurrence and two reported distant recurrence when the median follow-up time was set to be 43 months. Relapse-free survival, LC, and OS were 79%, 92%, and 74% at 3 years, respectively. This study suggested that locally advanced NPC can be treated more efficiently by PBT, but further trials are in process to enlighten the importance of this modality in improving the LC, life quality of patients after treatment, and OS.

Chondromas of the Base of the Skull and Chondrosarcomas (CS) ​​​​​

A chondroma is a benign tumor of the bone, whereas a CS is a malignant tumor involving the connective tissue. Since chondroma and CS of the base of the skull are at a sensitive spot due to the proximity to the brain, radical surgery and PT are not usually performed due to the overweighing risk of neurological dysfunction. Chondromas and CS are one of the earliest tumors to be treated with PBT due to their reduced toxicity on the adjacent organs. It began when Munzenrider and Liebsch conducted an interventional study where they performed PBT combined with IMRT on 519 patients with basilar skill chondroma and CS. Chondroma showed 78% survival, whereas CS showed 98% survival without local relapse and showed 11 cases of severe side effects, which is arguably acceptable [26]. Out of 11 patients, three faced severe side effects on the brainstem, while eight showed a varying degree of temporal lobe injuries. 

Later in 2005, Noel et al. conducted an interventional study on a cohort of 100 patients suffering from chondroma [27]. All the patients were given a median dose of 56 Gy of the proton beam, out of which 42% of patients reported noticeable late side effects. However, the researchers believed that the enhancements in the delivery method, precautions, and technique might improve the results significantly. 

Unilateral Tumors of the Head and Neck

Proton therapy is one of the best treatment modalities for HnN cancers that arise on only one side due to its near-zero exit dose. In 2013, Kandula et al. conducted a comparative study where they provided five patients with PT who had already received IMRT for their pre-existing unilateral HnN tumors [28]. It was noticed that median doses and normal tissue V10, V30, and V50 values were considerably lower in the proton plan. Hence, they concluded that PT has relatively lower toxic effects on adjacent critical organs of the head and neck. 

From 2011 to 2014, Romesser et al. conducted a comparative study on a group of 41 patients with one-sided cancer of a major salivary gland and squamous cell carcinoma [29]. Twenty-three of them were treated with IMRT, and 18 were treated with PT. In patients with IMRT, the plan exceeded the maximum median doses of the brainstem, spinal cord, oral cavity, contralateral parotid, and submandibular glands as compared to proton beam radiation therapy (PBRT). Moreover, PBRT was also associated with lower rates of acute toxicities with grade 2/+ acute dysgeusia 5.6% (65.2% in IMRT), mucositis 16.7% (52.2% in IMRT), and nausea 11.1% (56.5% in IMRT). They further concluded that PBRT is a preferable option for radiotherapy in patients with unilateral HnN cancers due to its normal tissue-sparing quality. 

Reirradiation for Recurring Cancers of the Head and Neck 

Reirradiation refers to salvage radiotherapy that overlaps with previously given radiotherapy. The primary purpose of reirradiation is to prevent painful death due to HnN cancers and reduce the local spread alongside improving OS. McDonald and his team conducted a clinical investigation where they treated 61 patients with proton reirradiation; 47.5% of the patients had had salvage surgery, whereas 52.5% out of 61 patients had squamous cell carcinoma and 16.4% had ACC [30]. The median dose of reirradiation was 66 Gy, and the median follow-up time was 15.2 months and 28.7 months for those who remained alive. They noticed a median OS of 16.5 months, whereas the two-year OS was 32.7%. Toxicities greater than grade 2 were seen in 14.7% in the early phase and in 24.6% in the late phase. One of the patients died within two months due to acute brainstem edema, which was likely caused by reirradiation. 

An investigation on recurrent NPC in 2019 involved 16 patients being treated with reirradiation PT. The median dose was 60 Gy, and the median follow-up was 10 months [31]. The researchers reported no acute grade 3 or higher toxicity, whereas 23.5% of patients presented with late ≥ 3-grade toxicities. The 18-month OS was reported to be 54.4%, and the local control was 66.6%. 

Unlike photon reirradiation, which comes with increased chances of severe toxicity, proton reirradiation in cases of recurrent HnN cancers is recommended due to its significantly reduced chances of toxicity and improved OS [32].

Effect of PT on the quality of life (QoL) of patients with HnN cancers 

The efficacy of PBRT in improving QoL compared to IMRT in HnN cancer patients has always been debated. Most of the studies that claim less toxicity of PT have not referred to the improvement in the QoL. However, few studies have shown improved QoL as compared to IMRT in patients with head and neck cancers following proton therapy. 

Srivastava et al. carried out a retrospective investigation in which 17 patients suffering from skull base chondroma and chondrosarcoma were treated with proton therapy over a period of six months [33]. They noticed a slight improvement in the QoL; however, those statistics and the sample size were insignificant. Therefore, the researchers concluded that it can be safely said that the QoL was at least maintained, if not improved, during the treatment of PT. 

Shih et al. conducted clinical trials in which they treated 20 patients suffering from grade 2 glioma with PT [34]. The patients received 54 Gy in 30 fractions and were assessed for QoL post treatment. The researchers claimed that no evidence supports the decline in patients’ QoL following PT. 

A comparative study by Sio et al. included patients suffering from oropharyngeal carcinoma [35]. Thirty-five of them were given chemotherapy and IMPT, while 46 were given chemotherapy and IMRT. They concluded that the patients who received IMPT with chemotherapy had a better QoL than those who received IMRT with chemotherapy. 

Given that, we concluded that PT provides a relatively better QoL post treatment than IMRT in patients with HnN cancer. However, more study is required to confirm the efficacy of PT on QoL in HnN cancer patients, as current research either has insignificant statistics or tiny sample sizes.

Downsides of proton therapy 

With all these abovementioned statistics, we can say that PBT’s effectiveness is far greater than that of IMRT, mainly in view of the side effect profile, but variations do exist, as mentioned earlier, and need further literature for the discovery of the benefits of PT. The major concern regarding the use of this modality is whether this technique can be employed for patient welfare on a large scale or not. If we take a look at all the trials that were conducted to assess its efficacy, it appears that the number of patients receiving this therapy was not that ample, based on which we can assign it as a treatment of choice for HnN cancers. 

Apart from sample sizes, cost-effectiveness has also emerged as an obstacle, as the establishment of proton therapy centers requires a substantial investment. Besides this, on clinical grounds, some approximations and uncertainties have become a hurdle in obtaining complete benefits out of this modality. As in the case of RBE, there’s a difference between its assumed value and that which is actually encountered. 

Future of proton therapy 

Besides its downsides, it can be safely estimated that PT is relatively one of the latest and safest advancements in the field of radiotherapy. Furthermore, recent advancements and enhancements in the PT delivery method, onboard imaging, and formulation of an automated proton plan have widened the use of PBT among radiologists. 

One of the major areas that require advancement in PBT is its cost-effectiveness. Nonetheless, PT is an emerging technology and hardly 25 years old. Therefore, it has a lot of room for further literature to be written and investigations to be carried out. For example, there is no adequate literature to support the effectiveness of PT to improve QoL in HnN cancer patients. Most of the literature that has addressed the QoL in HnN cancer patients post-PT either has a tiny sample size or insignificant statistics. 

## Conclusions

Our review shows that PBRT is a preferable mode of radiotherapy for HnN cancer patients as it targets the tumor cells with much more accuracy and spares normal tissues as compared to photon therapy. Proton beam radiation therapy has mainly lower incidents of acute and late toxicities. However, OS, LC, and QoL as compared to IMRT in HnN cancer patients are still debatable, and further research and randomized controlled trials are required for analyzing the efficacy of PBRT as the studies that are already conducted have either small sample sizes or are low levels of evidence. 
